# Viral load testing among women on ‘option B+’ in Mazowe, Zimbabwe: How well are we doing?

**DOI:** 10.1371/journal.pone.0225476

**Published:** 2019-12-03

**Authors:** Justice Nyakura, Hemant Deepak Shewade, Serge Ade, Angela Mushavi, Solomon Huruva Mukungunugwa, Anesu Chimwaza, Philip Owiti, Mbazi Senkoro, Owen Mugurungi

**Affiliations:** 1 AIDS and TB Unit, Ministry of Health and Child Care, Harare, Zimbabwe; 2 International Union Against Tuberculosis and Lung Disease (The Union), Paris, France; 3 The Union South-East Asia, New Delhi, India; 4 Karuna Trust, Bengaluru, Karnataka, India; 5 Faculté de Médecine, Université de Parakou, Parakou, Benin; 6 National Tuberculosis, Leprosy and Lung Disease Programme, Nairobi, Kenya; 7 National Institute for Medical Research- Muhimbili Medical Research Centre, Dar es Salaam, Tanzania; University of North Carolina at Chapel Hill, UNITED STATES

## Abstract

**Background:**

Globally, ten percent of new HIV infections are among children and most of these children acquire infection through mother-to-child transmission. To prevent this, lifelong ART among pregnant and breast feeding (PBF) women living with HIV, irrespective of the WHO clinical stage, was adopted (option B+). There is limited cohort-wise assessment of VL testing among women on ‘option B+’.

**Objective:**

Among a pregnancy cohort on antiretroviral therapy in public hospitals and clinics of Mazowe district, Zimbabwe (2017), to determine the i) proportion undergoing VL testing anytime up to six months post child birth and associated factors; ii) turnaround time (TAT) from sending the specimen to results receipt and VL suppression among those undergoing VL testing.

**Methods:**

This was a cohort study involving secondary programme data. Modified Poisson regression using robust variance estimates was used to determine the independent predictors of VL testing.

**Results:**

Of 1112 women, 354 (31.8%, 95% CI: 29.2–34.6) underwent VL testing: 113 (31.9%) during pregnancy, 124 (35%) within six months of child birth and for 117 (33.1%), testing period was unknown. Of 354, VL suppression was seen in 334 (94.4%) and 13 out of 20 with VL non-suppression underwent repeat VL testing. Among those with available dates (125/354), the median TAT was 93 days (IQR 19.3–255). Of 1112, VL results were available between 32 weeks and child birth in 31 (2.8%) women. When compared to hospitals, women registered for antenatal care in clinics were 36% less likely to undergo VL testing [aRR: 0.64 (95% CI: 0.53, 0.76)].

**Conclusion:**

Among women on option B+, the uptake of HIV VL testing was low with unacceptably long TAT. VL suppression among those tested was satisfactory. There is an urgent need to prioritize VL testing among PBF women and to consider use of point of care machines. There is a critical need to strengthen the recording and local utilisation of routine clinic data in order to successfully monitor progress of healthcare services provided.

## Introduction

HIV/AIDS remains a global health concern despite considerable resources that have been allocated in the past twenty years. Globally in 2017, 36.9 million people were living with HIV [1,2]. Since 2014, there has been a renewed commitment to fight HIV with the joint United Nations programme on HIV/AIDS (UNAIDS) launching the 90-90-90 targets with a view to end the HIV/AIDS epidemic by 2030 [3]. The third ‘90’ requires that by 2020, 90% of those on antiretroviral therapy (ART) are virally suppressed (<1000 copies per ml of plasma). In 2016, the World Health Organization (WHO) emphasized scaling up of viral load (VL) testing access in ART programmes in low- and middle-income countries, with pregnant and breastfeeding (PBF) women on ART being one of priority groups [4].

In 2017, 180 000 (10%) of the 1.8 million new HIV infections were amongst children [2]. More than 90% of children living with HIV acquire infection through mother-to-child transmission (MTCT) [5]. Half of all MTCT occurs during breastfeeding [6,7]. Unlike in general population, there is a limited window period during PBF period to institute HIV prevention interventions as each additional week of uncontrolled viremia increases MTCT risk [6,8]. The high risk stratification factors for MTCT are maternal VL over 1000 copies/ml at ≥32 weeks gestation, newly diagnosed HIV+ mothers during labour/delivery and breastfeeding period, incident infection (sero-conversion) during PBF, no ART or < 8 weeks of ART at delivery [4]. Therefore, it is important to understand the uptake and turnaround time (TAT) for VL testing in PBF women.

In 2017, access to VL testing remained low with a quarter of the countries reporting less than 50% uptake among those on ART [9]. Swannet et al noted 40% uptake among general population in Mozambique and factors associated with uptake included age, longer ART duration and differentiated service delivery services [10]. Among PBF women on ART, there is limited evidence on uptake and TAT for VL testing and VL suppression especially during breastfeeding. In Malawi, 84% VL suppression was reported in a randomised controlled trial among PBF women who underwent VL testing after six months of ART and longer ART duration was associated with viral suppression at child birth [11].

Zimbabwe, a low income country in Southern Africa and has a high HIV prevalence (14% among 15–49 years in general population). Since 2013, the country implemented option B+ for PBF women (lifelong ART among PBF women living with HIV irrespective of the WHO clinical stage) and since 2015, routine VL testing for monitoring of response to ART was initiated. In 2015–16, the population-based ‘ZIMPHIA’ survey reported that 60% of people living with HIV were virally suppressed (65% among women living with HIV) [12]. There is paucity of data regarding VL monitoring among PBF women in Zimbabwe. Hence, we conducted this operational research to assess the uptake and turnaround time (TAT) for VL testing amongst women on ‘option B+’ in a rural district in Zimbabwe in 2017. Additionally, we assessed the factors associated with HIV VL testing uptake.

## Methods

### Study design

This was a cohort study using routinely collected secondary data.

### Setting

#### General setting

Zimbabwe is a landlocked country divided into 10 provinces and 63 districts with a population of 13 million. Majority of the population (67%) lives in rural areas [13]. Approximately 95% of health facilities in Zimbabwe are public institutions providing both preventive and curative health services with free treatment available for HIV, TB, malaria and maternal and child health. According to the Zimbabwe demographic health survey in 2015, ninety three percent women aged 15–49 years received antenatal care (ANC) by a skilled provider and 72% of deliveries were conducted by a skilled provider [14]. In 2017, around 63 000 women living with HIV went for ANC and 90% of them were on ART [15].

Mazowe is a rural district in Mashonaland Central Province with a population of 0.23 million [13]. The public health facilities include three hospitals (one of them is district hospital) and 25 rural health clinics offering HIV prevention and treatment services (including prevention of mother to child transmission (PMTCT) services).

#### PMTCT programme

The ANC registration process involves manual recording of baseline demographic data of patients in the ANC register and issuing of a booking card. Child birth related details are filled in a delivery register. All pregnant women who are not living with HIV are offered HIV testing. Women found to be positive for HIV during ANC are initiated on ART, which is dispensed in ANC clinics with information being manually recorded in ANC register, ART register, and ART patient care booklet. ANC visits and ART visits are synchronised. For women already on ART, the ART patient care booklet is shifted from ART clinic to ANC clinic for a maximum period of two years post child birth. HIV exposed infant register is also maintained to record HIV prophylaxis, testing and follow up. The collected data from health facility registers is aggregated into a monthly return form that is sent to the district for data entry into the district health information system.

**Fig 1:**
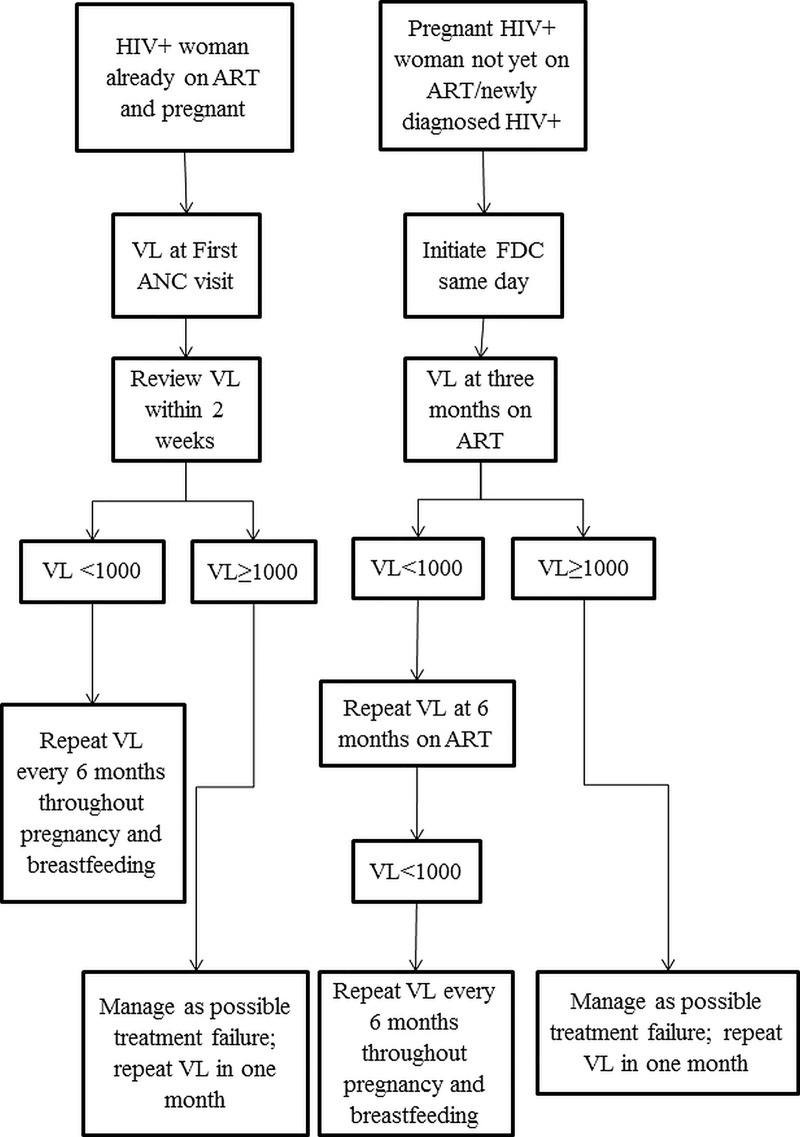
National algorithm for viral load testing among pregnant and breast feeding women living with HIV in Zimbabwe (2017). HIV–Human immunodeficiency virus; ART-Antiretroviral therapy; VL- Viral Load; FDC-Fixed dose combination; ANC-Antenatal care.

The national PMTCT programme mimics other national programmes in the region. The PMTCT programme has a national plan to eliminate MTCT of HIV and syphilis by 2022 [16]. The PMTCT programme provides a differentiated care service in the monitoring of VL among PBF women who are either ART naïve or already on ART. For ART naive PBF women, the first specimen for VL is taken after 3 months on ART. For those already on ART, if VL was not done within the last 6 months, it is done at the first ANC visit. If found to be virally non-suppressed, VL testing is repeated after conducting enhanced adherence counselling **(Fig 1)**.

Dried blood spot (DBS) and/or venous blood specimen (in ethylene diamine tetra-acetic acid vials) are sent to the national microbiology reference laboratory in Harare (maximum distance 72km) twice a week by a human carrier as a part of integrated specimen transportation. The results follow a similar route from testing laboratory back to the health facility. At facility level, VL register and ART care booklet are used to record date of specimen collection and VL result receipt.

### Patient population

Pregnant women living with HIV and having their first ANC visit in public health facilities in 2017 in Mazowe district were the patient population. The pregnancy cohort included pregnant women who were i) already on ART or ii) ART naïve at the time of first ANC visit and completed three months of ART.

### Data variables, sources of data and data collection

Between December 2018 and March 2019, data were collected from routine health facility registers (ANC register, ART booklet, delivery register and HIV exposed infant register) into a structured paper-based data collection form. Baseline characteristics included demographic data and antenatal care and HIV care related data. For ART naïve patients, information collected included date of HIV diagnosis and ART initiation. Dates of child birth, status of child and ART status of mother at six months post child birth, dates for VL testing and dates for VL result receipt by PBF women were obtained. Data on any VL results available at the facility during pregnancy and/or breastfeeding (up to six months post child birth) were collected. VL uptake was defined as when sample was collected and result was available at the clinic (irrespective of the result including invalid results).

### Analysis and statistics

Data were single-entered and analyzed using EpiData (version 4.1.1.0 for entry and version 2.2.2.183 for analysis, EpiData Association, Odense, Denmark). Adjusted analysis was performed using STATA (version 12.1 STATA Corp., College Station, TX, USA).

Baseline demographic, clinical and programmatic characteristics at ANC registration were summarized using frequency (proportion) and mean (standard deviation—SD) as applicable. Uptake of any VL test and availability of VL result after 32 weeks of gestation among the pregnancy cohort was reported using proportions (95% confidence interval (CI)).

The TAT in days from sending the specimen to receipt of result by PBF mother was summarised using median and interquartile range (IQR). Factors associated with uptake of VL test in the pregnancy cohort were summarized (inferred) using crude and adjusted relative risks (95% CI). Modified Poisson regression using robust variance estimates was used for the adjusted analysis. Factors with crude p<0.2 were included in the regression model.

### Ethics

Administrative approval for the study was obtained from Ministry of Health and Child Care AIDS and TB programme. Ethics approval was obtained from Medical Research Council of Zimbabwe (MRCZ/E/227, dated 06/12/2018) and The Union Ethics Advisory Group, Paris, France (EAG-55/18, dated 29/09/2018). As part of the ethics application, waiver for informed consent was sought and approved since the study involved review of secondary programme data.

## Results

### Baseline characteristics

**Fig 2:**
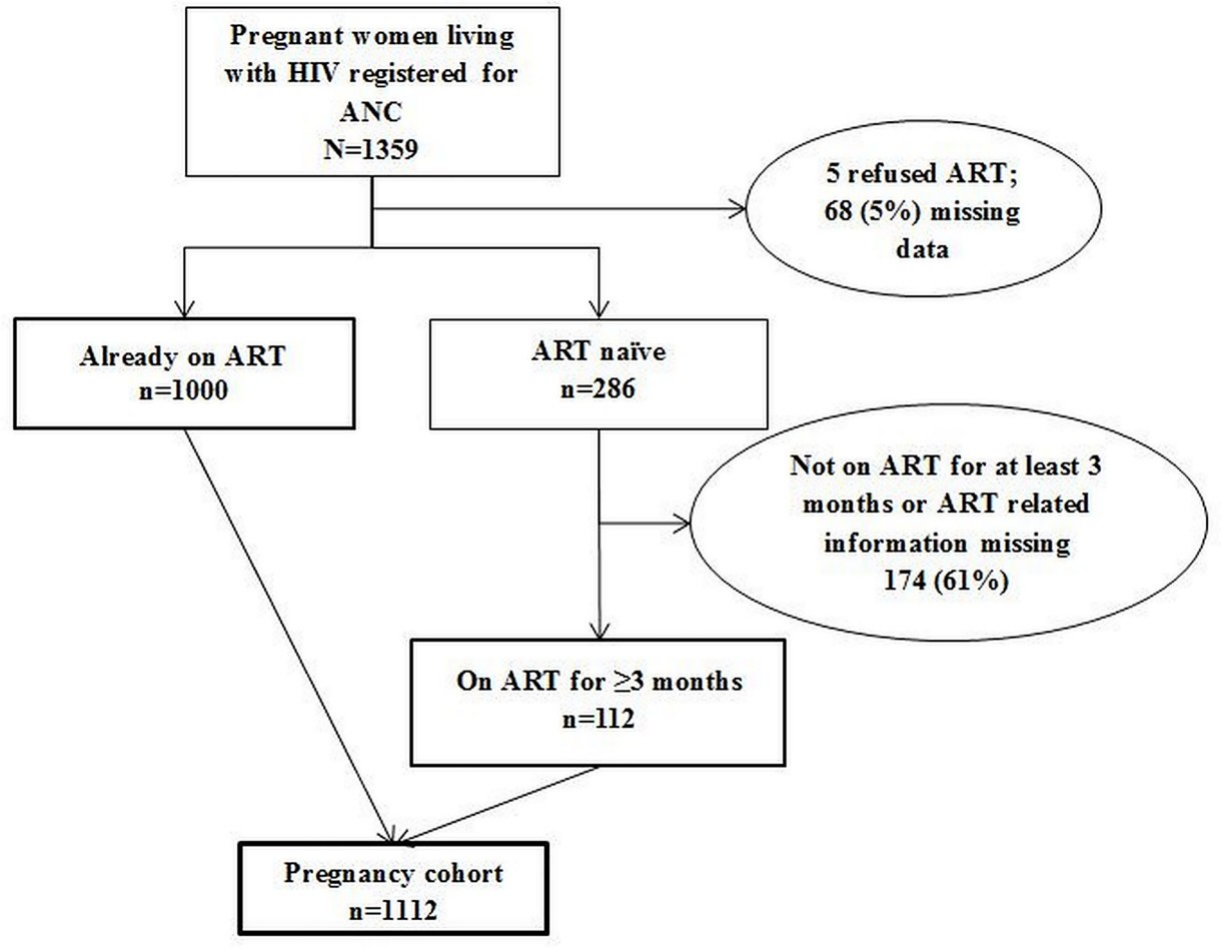
Flowchart describing the pregnancy cohort included in this study, Mazowe district, Zimbabwe (2017). VL–Viral load; ANC- Antenatal Care; ART-Antiretroviral treatment. In the pregnancy cohort, women already on ART were eligible for VL testing at ANC registration if VL results were not available in the previous six months. Women newly initiated on ART were eligible for first VL testing after completing three months of ART.

There were 1359 pregnant women living with HIV who registered for ANC in 2017 and of them 1112 formed the pregnancy cohort and were included in the study. There was missing data for 242 women **(Fig 2)**. The baseline characteristics of the pregnancy cohort are summarized in **Table 1**. Of 1112, a total of 112 (10%) were ART naïve whilst 1000 (90%) were already on ART at ANC registration. The mean (SD) age and gestational age in weeks at ANC registration was 30.3 (6.1) years and 18.8 (7.1) weeks, respectively.

**Table 1:** Demographic and clinical characteristics of pregnancy cohort (living with HIV) registered for ante-natal care in Mazowe District, Zimbabwe (2017).

Characteristics*			N	(%)
Total			1112	(100.0)
Age group (years)				
	15–19		46	(4.1)
	20–24		166	(14.9)
	25–29		283	(25.4)
	30–49		617	(55.5)
Gestational Age at ANC registration				
	= <12 weeks		197	(17.7)
	13–24 weeks		669	(60.2)
	25–32 weeks		171	(15.4)
	>32 weeks		44	(4)
	Missing		31	(2.8)
Gravida				
	1		78	(7)
	2		188	(16.9)
	3		294	(26.4)
	4		326	(29.3)
	>5		224	(20.1)
	Missing		2	(0.2)
Facility				
	Clinic		327	(29.4)
	Hospital		785	(70.6)
Anaemia**				
	No Anaemia		798	(71.8)
	Anaemia		111	(10)
	Severe Anaemia		6	(0.5)
	Missing		197	(17.7)
Hypertension (HTN)***				
	Normal		640	(57.6)
	Pre-HTN		355	(31.9)
	Stage 1 HTN		92	(8.3)
	Stage 2 HTN		13	(1.2)
	Missing		12	(1.1)
Weight (kgs)				
	30–44		20	(1.8)
	45–59		495	(44.5)
	> = 60		583	(52.4)
	Missing		14	(1.3)
ART Status				
	Already on ART		1000	(89.9)
	Newly Initiated		112	(10.1)
WHO Staging				
	Stage 1		208	(18.7)
	Stage 2		271	(24.4)
	Stage 3		98	(8.8)
	Stage 4		4	(0.4)
	Missing		531	(47.8)
Partner HIV Status				
	Positive		250	(22.5)
	Negative		209	(18.8)
	Unknown		653	(58.7)
Syphilis Test				
	Positive		56	(5)
	Negative		939	(84.4)
	Not recorded		117	(10.5)

*Characteristics as at first ANC visit

ANC–Antenatal care; ART–Antiretroviral therapy; HTN- Hypertension; WHO- World Health Organization

** No anaemia > = 11 hbg%; Anaemia = 7–11 hbg%; Severe Anaemia<7 hbg%

*** Normal is <120/80; pre-HTN is 120-139/80-89; Stage1 HTN is 140-159/90-109; Stage 2 HTN is > = 160/110

### VL testing

Of 1112 women, VL results were available for 354 (31.8%, 95% CI: 29.2–34.6). Of 354, a total of 334 (94.4%) were virally suppressed and 13 out of 20 virally non-suppressed women underwent repeat VL testing. Of 13, VL results were available for ten (nine were virally suppressed). Of 1112, VL results were available between 32 weeks’ gestation and child birth in 31 (2.8%, 95% CI: 1.9, 3.9) women.

**Fig 3:**
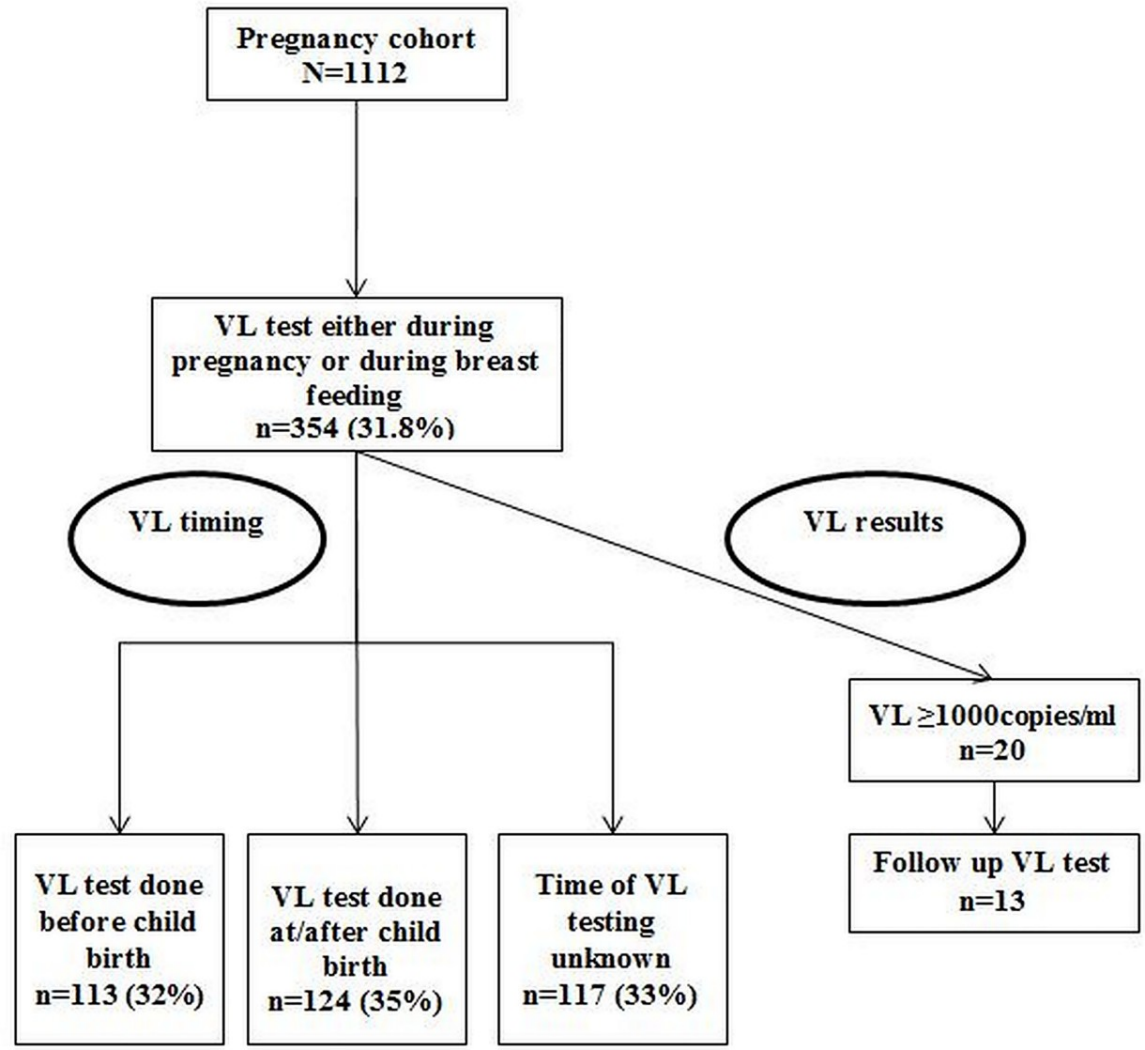
Flowchart describing the viral load testing among the pregnancy cohort (living with HIV and on ART) included in the study, Mazowe district, Zimbabwe (2017). VL- Viral load. Included those i) already on antiretroviral therapy before pregnancy and ii) newly diagnosed with HIV during antenatal care and on antiretroviral therapy for at least three months.

Of 354, 113 (31.9%) underwent testing during pregnancy, 124 (35%) within six months of child birth and for 117 (33.1%), dates were not available to classify when testing was done (**Fig 3**). Of 354, only 125 had the dates recorded to calculate median TAT. The median TAT from specimen collection to result receipt by PBF women was 93 days (IQR 19.3–255).

### Factors associated with uptake of VL testing

**Table 2** summarizes the factors associated with uptake of VL testing. On adjusted analysis, facility and WHO staging were significantly associated. When compared to hospitals, PBF women registered for ANC in clinics were 36% less likely to have a VL result [aRR: 0.64 (95% CI: 0.53, 0.76)]. When compared to WHO stage 1, women in stage 2 were 29% more likely to have a VL result [aRR: 1.29 (95% CI: 1.07, 1.55)].

**Table 2:** Factors associated with uptake of VL test among the pregnancy cohort (living with HIV and on ART) registered for ante-natal care in Mazowe district, Zimbabwe (2017).

Factors		Total	Viral Load uptake		RR	(95%CI)	aRR*	(95%CI)
		N	n	(%)				
Total		1112	354	(31.8)				
Age group (years)								
	15–19	46	8	(17.4)	0.50	(0.26, 0.94)^	0.70	(0.35,1.43)
	20–24	166	46	(27.7)	0.79	(0.60,1.03)	0.79	(0.59,1.05)
	25–29	283	83	(29.3)	0.83	(0.68,1.03)	0.95	(0.78,1.17)
	30–49	617	217	(35.2)	Ref			
Gestational Age at ANC registration								
	= <12 weeks	197	57	(28.9)	Ref			
	13–24 weeks	669	227	(33.9)	1.17	(0.92,1.50)	0.97	(0.79,1.18)
	25–32 weeks	171	46	(26.9)	0.93	(0.67,1.29)	0.85	(0.64,1.12)
	>32 weeks	44	9	(20.5)	0.71	(0.38,1.32)	0.67	(0.37,1.19)
	Missing	31	15	(48.4)	1.67	(1.09,2.56)^	1.41	(0.90,2.23)
Gravida								
	1	78	18	(23.1)	0.57	(0.37,0.89)^	0.81	(0.48,1.34)
	2	188	53	(28.2)	0.70	(0.53,0.93)^	0.93	(0.70,1.22)
	3	294	96	(32.7)	0.81	(0.65,1.02)	1.07	(0.87,1.31)
	4	326	96	(29.4)	0.73	(0.58,0.92)^	0.90	(0.74,1.09)
	>5	224	90	(40.2)	Ref			
	Missing	2	1	(50.0)	----		1.25	(0.82,1.91)
Facility								
	Clinic	785	208	(26.5)	0.59	(0.50,0.70)^	0.64	(0.53,0.76)^
	Hospital	327	146	(44.6)	Ref			
Anaemia**								
	No Anaemia	798	253	(31.7)	Ref			
	Anaemia	111	50	(45.0)	1.42	(1.13,1.79)^	1.08	(0.87,1.34)
	Severe Anaemia	6	1	(16.7)	0.53	(0.09,3.16)	0.59	(0.20,1.70)
	Missing	197	50	(25.4)	0.80	(0.62,1.04)	0.84	(0.67,1.07)
Hypertension (HTN) ***								
	Normal	640	196	(30.6)	Ref		†	
	Pre-HTN	355	123	(34.6)	1.13	(0.94,1.36)		
	Stage 1 HTN	92	28	(30.4)	0.99	(0.71,1.38)		
	Stage 2 HTN	13	5	(38.5)	1.26	(0.63,2.52)		
	Missing	12	2	(16.7)	0.54	(0.15,1.94)		
Weight (kgs)								
	30–44	20	6	(30.0)	0.96	(0.48,1.89)	†	
	45–59	495	161	(32.5)	1.04	(0.87,1.23)		
	> = 60	583	183	(31.4)	Ref			
	Missing	14	4	(28.6)	0.91	(0.39,2.10)		
ART Status								
	Already on ART	1000	307	(30.7)	0.73	(0.58,0.93)^	0.98	(0.76,1.25)
	Newly Initiated	112	47	(42.0)	Ref			
WHO Staging								
	Stage 1	208	97	(46.6)	Ref			
	Stage 2	271	159	(58.7)	1.26	(1.05,1.50)^	1.29	(1.07,1.55)^
	Stage 3	98	59	(60.2)	1.29	(1.04,1.60)^	1.19	(0.96,1.49)
	Stage 4	4	1	(25.0)	0.54	(0.10,2.95)	0.54	(0.12,2.51)
	Missing	531	38	(7.2)	0.15	(0.11,0.22)^	0.15	(0.11,0.22)^
Partner HIV Status								
	Positive	250	82	(32.8)	Ref			
	Negative	209	54	(25.8)	0.79	(0.59,1.05)	0.83	(0.64,1.09)
	Unknown	653	218	(33.4)	1.02	(0.83,1.25)	0.94	(0.78,1.13)
Syphilis Test								
	Positive	56	17	(30.4)	Ref			
	Negative	939	294	(31.3)	1.03	(0.69,1.55)	†	
	Not recorded	117	43	(36.8)	1.21	(0.76,1.92)		

Row percentage. ANC–Antenatal Care; HTN- Hypertension; ART–Antiretroviral therapy; WHO- World Health Organization; RR–relative risk; aRR–adjusted relative risk; CI- confidence interval

* Adjusted analysis conducted using modified Poisson regression

† weight, hypertension and syphilis test were not included in the regression model as their unadjusted p value was >0.2

**Anaemia—No anaemia > = 11 hbg%; Anaemia = 7–11 hbg%; Severe Anaemia<7 hbg%

*** Hypertension—Normal is <120/80; pre-HTN is 120-139/80-89; Stage1 HTN is 140-159/90-109; Stage 2 HTN is > = 160/110

^p<0.05

### Missing data

Of 1112 women in pregnancy cohort, as per information available in the records, we could assign only 453(40.7%) to the breastfeeding cohort (mother on ART and child alive at six months post child birth). There were various reasons for this which include missing data for delivery date [n = 631 (56.7%)] and ART outcome at six months post child birth [n = 629 (56.6%)]. Of 354 VL results available, dates for sending VL specimen, receipt of results at facility and receipt of results by PBF women were missing in 49(13.8%), 328 (92.7%) and 191(54.0%) instances, respectively.

## Discussion

This is one of the first cohort-wise assessments of HIV VL testing amongst PBF women living with HIV in programmatic settings. In a rural district of Zimbabwe, we found poor data documentation in the routine registers, low testing uptake and long turnaround times. Most of them with a result were virally suppressed.

### Key findings

The study has many programme relevant findings. First, there was poor routine data documentation, almost half of the records had missing data. We could not report VL uptake separately for the pregnancy and breast feeding cohort as we did not have the correct denominator to distinguish the two groups due to missing dates. In almost half of the records, we observed missing delivery date, date VL specimen taken and date results were received by the PBF woman. We could not provide the breakdown of the TAT for VL testing due to missing dates. The ART start date was missing for many pregnant women who were already on ART. Hence, we could not analyse the association between duration of ART and VL testing. In a PMTCT study done in Harare city, Zimbabwe, 90% of study population lacked adequate information to determine whether mother belonged to the high risk MTCT group or not [17].

Second, VL testing uptake was low with unacceptably long turnaround times. Long TATs prevent effective utilization of VL results for clinical decision making. It is very much possible that long TATs can in turn have a negative effect on VL testing uptake. In low- and middle-income countries, low uptake of healthcare services and adherence to treatment and monitoring guidelines is affected by fragile healthcare systems and lack of resources [18]. Similar to our study, in Kenya, VL specimen and test results required distant shipments from the clinics to the testing laboratory and back [19].

The VL testing uptake was lower when compared to other studies in the region. In programmatic setting in Mozambique, VL testing uptake was 40% in general population [10]. Among pregnant women living with HIV in a predominantly rural setting in Swaziland, the VL uptake was 67% [20]. The Swaziland study was a pilot on the feasibility of option B+ with specially trained staff that included mobile medical doctors attending to clinically complicated patients. Therefore the circumstance were different from our study which dealt with routinely collected data [21,22].

Most studies report TAT from specimen collection to processing time in the laboratory or intra-laboratory TAT. A study in Kenya reported TAT of 21 days from specimen collection to results dispatch from laboratory, whilst a study in Malawi reported intra-laboratory TAT of 18 days [23,24]. This may not be comparable with our study as we reported TAT from specimen collection at facility to result receipt by PBF women (this includes receipt at laboratory, dispatch results and receipt of results at the facility).

Third, satisfactory viral suppression in our study (higher than UNAIDS target of 90% for all women on ART) should be interpreted with caution as majority of PBF women did not undergo VL testing. This might not be the true picture of VL suppression among those on ART. Viral suppression of 84% in Malawi (from a randomized controlled trial) and 85% in rural South Africa (programmatic setting) should also be interpreted with caution as the VL testing uptake was not reported [11,25].

Fourth, we found a significantly lower uptake of VL testing in clinics as compared to hospitals. Shortage of trained staff, poor working conditions, low staff morale and poor management have been implicated for low uptake of healthcare services in remote rural areas in Sub Saharan Africa [22,26]. Recent initiatives such as quality improvement have improved provision and uptake of ANC services in hard to reach areas [22,27]. In clinics in rural Kenya, quality improvement approaches increased adherence to standards of ANC from <40% to 80–100% [22]. Similarly, rural clinics in Zimbabwe can be prioritised for quality improvement initiatives for PMTCT indicators.

### Implications for policy and practice

There is a need to prioritize VL testing in PBF women through improved monitoring and supervision. The first step is to urgently improve the completeness of routine data collected. Every healthcare worker at the health facility should be trained in routine data collection. This activity can ride on the existing PMTCT and ART mentoring programme. The routine data collection tools can also be introduced as part of pre-service training for nurses as they man primary care facilities in Zimbabwe.

Additionally, the reports of quarterly supportive supervision conducted by district management in selected facilities should be utilized to improve the data quality in all the facilities of the district. The tools used for support and supervision conducted by the district team should include assessment on data completeness, timeliness, accuracy and consistency in the registers.

The submission of monthly return form has been successful with completion rates consistently above 95%, this is because there is proactive monitoring and follow up of these forms since they are part of the nationally reported indicators. In addition to already present indicators in the monthly return form [number of pregnant women living with HIV newly registered for ANC that are ART naïve (a); and number of pregnant women living with HIV newly registered for ANC that are already on ART (b)], there is a need to add another indicator ‘number of VL specimen of pregnant women sent (c)’. At the district, provincial and national level, VL testing uptake during pregnancy can be reviewed using the formula ‘c ≥ 0.85*a + 0.5*b’ (assuming 85% of ART naïve pregnant women are retained in ART care at three months and 50% of pregnant women already on ART do not have a VL result in last six months). If ‘c’ is less than ‘0.85*a + 0.5*b’, feedback should be sent and corrective actions taken. Similarly, testing uptake during breast feeding may be reviewed.

Second, point of care machines or information technology to transmit the results is urgently required to lower the TAT. For early infant diagnosis of HIV in Zimbabwe, point of care assay has been found to be cost-effective than conventional testing [28]. The same may be applicable for VL testing as well. The country can leverage on the already available point of care devices in some rural clinics to offer HIV VL testing.

Finally, there is an urgent need for qualitative systematic enquiry to understand the possible reasons and suggested solutions from the user perspective (PBF mother and facility staff).

### Limitations

There were two limitations. In addition to the missing data which is a key limitation, the study did not look at health systems related factors and other socio-demographic characters which can affect service uptake. This was not routinely collected and therefore, beyond the scope of this study.

## Conclusion

There is a critical need to strengthen the recording and local utilisation of routine clinic data in order to successfully monitor progress of healthcare services provided.VL testing uptake was low with turnaround times unacceptably long among PBF women on ‘option B+’ in Mazowe district, Zimbabwe. Viral load suppression was satisfactory among those tested. There is an urgent need to prioritize VL monitoring in PBF women and consider use of point of care assays. These activities are urgently needed if Zimbabwe is to meet the 2022 national target of reducing MTCT to less than five percent.

### Disclosure statement

None of the authors have any competing interest. The contents of this paper do not necessarily reflect the views of the Government or Non-Governmental Organizations or Academic institutions or The Union.

## References

[1] Dieleman JL, Singh L, Birger M, Schneider M, Chapin A. Tracking development assistance for HIV/AIDS by type of investment, 1990–2015. Lancet Glob Heal. 2016;4: S35 10.1016/S2214-109X(16)30040-7 PMC4867985 26950317

[2] United Nations programme on HIV/AIDS. UNAIDS Data 2018. Geneva, Switzerland; 2018.

[3] United Nations programme on HIV/AIDS. Fast track: Ending the AIDS epidemic by 2030. Geneva, Switzerland; 2014.

[4] World Health Organisation. Consolidated Guidelines on the Use of Antiretroviral Drugs for Treating and Preventing HIV Infection: Recommendations for a Public Health Approach. 2nd Edition. Geneva, Switzerland; 2016. 27466667

[5] United Nations programme on HIV/AIDS. UNAIDS report on the global AIDS epidemic 2010. Geneva, Switzerland: World Health Organization; 2010.

[6] Myer L, Essajee S, Broyles LN, Watts DH, Lesosky M, El-Sadr WM, et al Pregnant and breastfeeding women: A priority population for HIV viral load monitoring. PLOS Med. 2017;14: e1002375 10.1371/journal.pmed.1002375 28809929 PMC5557351

[7] United Nations programme on HIV/AIDS. Get on the Fast-Track:The life-cycle approach to HIV. Geneva Switzerland; 2016.

[8] Townsend CL, Byrne L, Cortina-Borja M, Thorne C, de Ruiter A, Lyall H, et al Earlier initiation of ART and further decline in mother-to-child HIV transmission rates, 2000–2011. AIDS. 2014;28: 1049–57. 10.1097/QAD.0000000000000212 24566097

[9] United Nations programme on HIV/AIDS. Knowledge is Power Know Your Status, Know Your Viral Load. Geneva, Switzerland; 2018.

[10] Swannet S, Decroo T, de Castro SMTL, Rose C, Giuliani R, Molfino L, et al Journey towards universal viral load monitoring in Maputo, Mozambique: many gaps, but encouraging signs. Int Health. 2017;9: 206–214. 10.1093/inthealth/ihx021 28810670 PMC5881256

[11] Hosseinipour M, Nelson JAE, Trapence C, Rutstein SE, Kasende F, Kayoyo V, et al Viral Suppression and HIV Drug Resistance at 6 Months Among Women in Malawi’s Option B+ Program: Results From the PURE Malawi Study. J Acquir Immune Defic Syndr. 2017;75 Suppl 2: S149–S155. 10.1097/QAI.0000000000001368 28498184 PMC5431274

[12] Ministry of Health and Child Care. Zimbabwe Population Based HIV Impact Assessment. Harare; 2017.

[13] Zimbabwe Statistical Agency. Census 2012, National Report. Harare, Zimbabwe; 2012.

[14] Zimbabwe Statistical Agency, The DHS Program ICF International. Zimbabwe Demographic and Health Survey. Harare, Zimbabwe; 2015.

[15] Ministry of Health and Child Care. AIDS &TB Report 2017. Harare, Zimbabwe; 2018.

[16] Ministry of Health and Child Care. The Plan for Elimination of Mother to Child Transmission of HIV & Syphilis in Zimbabwe 2018–2022. Harare, Zimbabwe; 2017.

[17] Komtenza B, Satyanarayana S, Takarinda KC, Mukungunugwa SH, Mugurungi O, Chonzi P, et al Identifying high or low risk of mother to child transmission of HIV: How Harare City, Zimbabwe is doing? PLoS One. 2019;14: e0212848 10.1371/journal.pone.0212848 30865646 PMC6415877

[18] Gamell A, Letang E, Jullu B, Mwaigomole G, Nyamtema A, Hatz C, et al Uptake of guidelines on prevention of mother-to-child transmission of HIV in rural Tanzania: time for change. Swiss Med Wkly. 2013;143: w13775 10.4414/smw.2013.13775 23519621

[19] Awungafac G, Amin ET, Fualefac A, Takah NF, Agyingi LA, Nwobegahay J, et al Viral load testing and the use of test results for clinical decision making for HIV treatment in Cameroon: An insight into the clinic-laboratory interface. PLoS One. 2018;13: e0198686 10.1371/journal.pone.0198686 29889862 PMC5995384

[20] Etoori D, Kerschberger B, Staderini N, Ndlangamandla M, Nhlabatsi B, Jobanputra K, et al Challenges and successes in the implementation of option B+ to prevent mother-to-child transmission of HIV in southern Swaziland. BMC Public Health. 2018;18: 374 10.1186/s12889-018-5258-3 29558896 PMC5859825

[21] Yeap AD, Hamilton R, Charalambous S, Dwadwa T, Churchyard GJ, Geissler PW, et al Factors influencing uptake of HIV care and treatment among children in South Africa–a qualitative study of caregivers and clinic staff. AIDS Care. 2010;22: 1101–1107. 10.1080/09540121003602218 20824563

[22] Mwaniki MK, Vaid S, Chome IM, Amolo D, Tawfik Y, Kwale Improvement Coaches. Improving service uptake and quality of care of integrated maternal health services: the Kenya Kwale District improvement collaborative. BMC Health Serv Res. 2014;14: 416 10.1186/1472-6963-14-416 25240834 PMC4179240

[23] Mwau M, Syeunda CA, Adhiambo M, Bwana P, Kithinji L, Mwende J, et al Scale-up of Kenya’s national HIV viral load program: Findings and lessons learned. PLoS One. 2018;13: e0190659 10.1371/journal.pone.0190659 29324811 PMC5764307

[24] Minchella PA, Chipungu G, Kim AA, Sarr A, Ali H, Mwenda R, et al Specimen origin, type and testing laboratory are linked to longer turnaround times for HIV viral load testing in Malawi. PLoS One. 2017;12: e0173009 10.1371/journal.pone.0173009 28235013 PMC5325555

[25] Chetty T, Newell M-L, Thorne C, Coutsoudis A. Viraemia before, during and after pregnancy in HIV-infected women on antiretroviral therapy in rural KwaZulu-Natal, South Africa, 2010–2015. Trop Med Int Heal. 2018;23: 79–91. 10.1111/tmi.13001 29121445 PMC7612915

[26] Pearson L, Shoo R. Availability and use of emergency obstetric services: Kenya, Rwanda, Southern Sudan, and Uganda. Int J Gynaecol Obstet. 2005;88: 208–15. 10.1016/j.ijgo.2004.09.027 15694109

[27] Sarin E, Kole SK, Patel R, Sooden A, Kharwal S, Singh R, et al Evaluation of a quality improvement intervention for obstetric and neonatal care in selected public health facilities across six states of India. BMC Pregnancy Childbirth. 2017;17: 134 10.1186/s12884-017-1318-4 28464842 PMC5414154

[28] Frank SC, Cohn J, Dunning L, Sacks E, Walensky RP, Mukherjee S, et al Clinical effect and cost-effectiveness of incorporation of point-of-care assays into early infant HIV diagnosis programmes in Zimbabwe: a modelling study. lancet HIV. 2019;6: e182–e190. 10.1016/S2352-3018(18)30328-X 30737187 PMC6408227

